# Attributes of Oct4 in stem cell biology: perspectives on cancer stem cells of the ovary

**DOI:** 10.1186/1757-2215-5-37

**Published:** 2012-11-21

**Authors:** Chantel Samardzija, Michael Quinn, Jock K Findlay, Nuzhat Ahmed

**Affiliations:** 1 Women’s Cancer Research Centre, Royal Women’s Hospital, 20 Flemington Road, Parkville, VIC, 3052, Australia; 2 Department of Obstetrics and Gynaecology, University of Melbourne, Melbourne, VIC, 3052, Australia; 3 Prince Henry’s Institute of Medical Research, Melbourne, VIC, 3168, Australia

**Keywords:** Ovarian carcinoma, Cancer stem cell, Metastasis, Chemoresistance, Recurrence, Embryonic stem cells, Induced pluripotent stem cells

## Abstract

Epithelial ovarian cancer (EOC) remains the most lethal of all the gynaecological malignancies with drug resistance and recurrence remaining the major therapeutic barrier in the management of the disease. Although several studies have been undertaken to understand the mechanisms responsible for chemoresistance and subsequent recurrence in EOC, the exact mechanisms associated with chemoresistance/recurrence continue to remain elusive. Recent studies have shown that the parallel characteristics commonly seen between embryonic stem cells (ESCs) and induced pluripotent stem cells (iPSC) are also shared by a relatively rare population of cells within tumors that display stem cell-like features. These cells, termed ‘cancer initiating cells’ or ‘cancer stem cells (CSCs)’ have been shown not only to display increased self renewal and pluripotent abilities as seen in ESCs and iPSCs, but are also highly tumorigenic in in vivo mouse models. Additionally, these CSCs have been implicated in tumor recurrence and chemoresistance, and when isolated have consistently shown to express the master pluripotency and embryonic stem cell regulating gene Oct4. This article reviews the involvement of Oct4 in cancer progression and chemoresistance, with emphasis on ovarian cancer. Overall, we highlight why ovarian cancer patients, who initially respond to conventional chemotherapy subsequently relapse with recurrent chemoresistant disease that is essentially incurable.

## Introduction

Ovarian cancer is the fifth leading cause of cancer-related death in women worldwide and has the highest rate of cancer-related mortality among all the gynaecological neoplasms in the Western world [1]. It predominately affects postmenopausal women, with approximately 204,000 women diagnosed with this disease each year [2]. While the term ‘ovarian cancer’ encompasses a broad range of ovarian neoplasms [3], there are three basic classes of ovarian malignancies and each arise from the rapid growth and division of one of three major cell types found within the ovary [4]. Tumors developing from the oocyte producing germ cells are known as germ cell tumors (~5-10%), while those arising from specialised granulosa, theca and hilus cells are classified as sex cord stromal tumors (~10-15%) [4]. In comparison however, these tumors represent a relatively rare group of ovarian malignancies, with cancers of the ovarian surface epithelium accounting for the largest proportion of all ovarian cancer cases. Such tumors have been termed Epithelial Ovarian Cancers (EOCs) and account for 90% of all ovarian tumors [4]. Despite an improved knowledge about EOCs and advances in existing treatments, more than 70% of EOC patients succumb to the disease within 5 years of their initial diagnosis [5], contributing annually to more than 125,000 deaths worldwide [2]. If however the disease is detected at an early stage, the five year survival rate dramatically increases to 95% [1]. Unfortunately, the asymptomatic nature of the disease combined with the lack of specific screening techniques for early detection, means most women are diagnosed when the disease has progressed to an advanced metastatic stage (Stages III-IV) [1].

### Progression of epithelial ovarian cancer

Advanced stage EOC is a highly metastatic disease commonly characterised by widespread peritoneal dissemination and ascites [6]. Due to the lack of anatomical barriers surrounding the ovaries, ovarian carcinomas are easily capable of disseminating directly from the ovary and into the peritoneal cavity, thus allowing the direct attachment of malignant cells to the peritoneum to form secondary tumors. This occurs by either mechanical or enzymatic disruption where malignant cells are then able to form cellular aggregates (spheroids) within the peritoneal ascites fluid [6,7]. As a result, the spheroids are capable of attaching and infiltrating the mesothelial lining of the peritoneal cavity resulting in the formation of secondary tumors [7,8]. Currently, this presents a major problem in the treatment of ovarian cancer as cytoreductive surgery is unable to completely eliminate microscopic disease within the peritoneum [6].

### Chemoresistance in epithelial ovarian cancer

Frontline treatment of advanced EOC usually involves cytoreductive surgery followed by systemic administration of chemotherapy [5,6]. Currently, the standard chemotherapy regime is a combination of a platinum compound such as cisplatin or carboplatin synergised with a taxol-based agent, usually paclitaxel [5,6]. Overall, this has achieved an initial patient response rate of 70-80% [5]. However, even in patients who initially respond well, most relapse in a relatively short period of time and the overall prognosis continues to remain poor [5]. This can be partly attributed to the advanced-stage of the disease at diagnosis and the highly aggressive nature of the disease. However, one of the most important causes of failure in EOC treatment is the development of residual and recurrent tumor cells that are resistant to cisplatin and paclitaxel treatment [6]. It has recently been shown that while platinum-based treatments are extremely efficient in removing the bulk of the ovarian tumor mass, it fails to eliminate a core of highly specialised CSC-like cells, which are not only highly invasive but are capable of initiating new tumor growth [9,10]. Recurrent ovarian tumors are known to be enriched with CSC-like cells and stem cell pathway mediators including ALDH1, CD44, CD133, Notch, Wnt and TGFβ, suggesting that CSCs may contribute to recurrent disease [11].

### Oct4 and its role as an embryonic stem cell factor in adult tissues

Oct4 (Oct3/4 or POU5F1) is a member of the POU family of transcription factors and is known to play a pivotal role in the maintenance of self-renewal and pluripotency in ESCs. It is commonly expressed in unfertilized oocytes, the inner cell mass (ICM) of a blastocyst, germ cells, embryonic carcinoma cells and embryonic germ cells [12]. While upregulation of Oct4 sustains an undifferentiated pluripotent stem cell state, a loss of Oct4 induces stem cells to undergo differentiation, producing a heterogenous population of highly specialised daughter cells. This is evidenced by the loss of pluripotency in the ICM cells of Oct4^−/−^ mouse embryos, where loss of Oct4 results in the differentiation of embryonic stem cells into a trophoblast lineage [13]. Studies have also demonstrated that a two-fold increase in Oct4 expression results in the conversion of ESCs towards a primitive endoderm and mesoderm state [14]. Conversely, a 50% decrease in Oct4 expression can induce differentiation of ESC into trophectoderm [14]. This suggests that the precise level of Oct4 protein expression in ESCs is crucial to maintain lineage-specific ESC differentiation and different developmental fates. Little is known about the exact regulation of Oct4 protein in ESCs, although it has been suggested that a highly sensitive sensor mechanism exists that is capable of detecting and regulating Oct4 levels within ESCs [12].

Interestingly, although Oct4 is primarily expressed in primitive ESCs and its expression is lost with differentiation during the developmental process [15], it has been shown that a minor population of very small embryonic-like stem cells (VSELs) with pluripotent potential and positive for Oct4, stage specific embryonic antigen-(SSEA)-3/4 (human), Sca-1 and Nanog [15,16] are present in the bone marrow, cord blood, epidermis, heart, pancreas, testis, bronchial epithelium and ovaries [15,17]. It has been hypothesized that VSELs expressing both epiblast and germ line markers are deposited in developing tissues and organs during early gastrulation [18]. The Oct4 promoter in these cells has been shown to have an open chromatin structure which can be actively transcribed, suggesting the transcription abilities of these cells [19]. However, these cells are protected from uncontrolled proliferation and teratoma formation by a unique DNA methylation pattern in some developmentally crucial imprinted genes which show a hypomethylation pattern in paternally methylated genes [insulin-like growth factor 2 (Igf2) and Rasgrf1] and hypermethylation in the maternally methylated genes [of H19, Igf2 receptor (igf2R) and p57Kip2 (also known as Cdkn 1c)] [18,19]. It has been demonstrated that, reversal of these epigenetic changes in VSELs may result in a greater expansion of these cells [18], and a few recent studies have demonstrated that both murine and human Oct4 positive VSELs exhibit characteristics of long-term repopulating hematopoetic stem cells [20] and may also differentiate into organ-specific cells (such as cardiomyocytes) [21]. Consistent with that, a gradual decrease in the number of Oct4 positive VSELs has been shown to be an important mechanism of aging, as evidenced in a recent murine model [22]. These results suggest that isolated Oct4 positive VSELs may serve as a good source of pluripotent stem cells in adult tissues and have a potential application in regenerative medicine [16]. It has been hypothesized that in pathological conditions the tissue-specific VSELs may undergo mutation in the ‘quiescence associated genetic imprints’ which may initiate the development of tissue-specific malignancies [18].

### Oct4 and its role as an induced pluripotent stem cell factor

The past decade has shown that somatic cells can be reprogrammed into induced pluripotent stem cells (iPSCs) by transient ectopic expression of a cocktail of transcription factors such as Oct4, Sox2, Klf4 and c-Myc [23-25]. For example, human fibroblasts can be reprogrammed by the ectopic expression of Oct4, Sox2, Nanog and Lin28 [26], or similarly by the over expression of only Oct4 and Bmi1 [27]. To date, iPSCs have been derived from numerous human somatic cell populations and closely resemble human ESCs in gene expression, promoter methylation and differentiation potential [28]. However, among the several combinations of transcription factors needed to make iPSC, Oct4 is the only one that has been shown to be required exogenously, suggesting that this transcription factor may act as a ‘gatekeeper’ of pluripotency in somatic cells [29]. Nonetheless, some recent studies have shown that while Oct4 can be replaced by the overexpression of E-cadherin or the orphan nuclear receptor Nr5a2 together with Sox2, Klf4 and c-Myc for reprogramming of mouse embryonic fibroblasts, the reprogramming efficiency decreases when Oct4 is not present [30,31], emphasizing once again the absolute requirement of Oct4 for the efficient reprogramming of somatic cells into iPSCs.

### Synergies of cancer cells with ESC and iPSC in the context of Oct4 expression

Cancer cells exhibit traits that are commonly associated with ESCs or iPSCs including immortal cell growth and high proliferation rates under appropriate culture conditions [32]. Both iPSCs and cancer cells are characterised as having high telomerase activity [33] and genomic instability resulting in chromosomal aberrations [34]. Changes in gene expression profiles and corresponding epigenetic changes have been observed in cancer cells, ESCs and iPSCs [35-37]. Additionally, similar to cancer cells, both ESCs and iPSCs give rise to teratomas after transplantation into immunocompromised mice [38]. In this context it should be mentioned that the common reprogramming factors such as c-Myc, Klf4, Sox2, Lin28 and Oct4 are highly expressed in many cancer cell types [39,40], suggesting that reprogramming of somatic cells and tumorigenesis rely on common mechanisms.

### Role of Oct4 in tumor progression

The first involvement of Oct4 in cellular transformation was observed when ectopic dose-dependent expression of Oct4 was shown to increase the malignant potential of ESCs [41]. Exogenous expression of Oct4 has been shown to mitigate dysplasia in the epithelial tissues of adult mice [42]. Consistent with that, Oct4 is expressed in a number of malignant neoplasms and the expression profile has been correlated with tumor grade and disease progression [43-46]. Compared to tumors with low Oct4 expression, elevated levels of Oct4 have been associated with metastases and shorter patient survival rates, [47-49]. A recent study on breast cancer has demonstrated that ectopic expression of Oct4 in normal breast cells led to the generation of cells with tumor-initiating and colonization abilities [50]. These cells developed high-grade, poorly differentiated breast carcinomas in nude mice and demonstrated a genomic profile enriched in an embryonic transcription factor network, suggesting that Oct4-transduced cells may represent a patient-specific model system for the discovery of novel oncogenic targets [50]. Furthermore, ectopic Oct4 expression has been shown to enhance the features of cancer stem cells in a mouse model of breast cancer [51]. Oct4 expression has also been shown to maintain CSC-like properties in CD133-derived lung cancer cells [52]. Overall, these studies highlight the importance of sustaining Oct4 expression by tumors in order to maintain the tumorigenic stem cell-like characteristics.

Epithelial to mesenchymal transition (EMT) is a vital process for morphogenesis during embryonic development [53], and also for the conversion of early stage tumors to invasive neoplasms [54]. Recent studies have demonstrated that EMT also plays a critical role in tumor recurrence which is believed to be tightly linked with the CSC phenotype [55,56]. A recent study on prostate cancer has demonstrated that prostate cancer cell lines that acquired EMT phenotype shared a stem cell-like signature including enhanced expression of Oct4 and increased tumorigenicity in mice [57]. In addition, ectopic expression of Oct4 and Nanog in lung adenocarcinoma cell line has been shown to increase the percentage of sub-population cells expressing CD133, drug resistance and promote EMT [58]. In contrast, down regulation of Oct4 in a breast cancer cell line which has a high endogenous level of Oct4 has been shown to promote invasion and metastasis by inducing EMT [59]. These contradictory results suggest that the reprogramming-competent Oct4 can differentiate cancer cells to either an epithelial or mesenchymal state of plasticity. In these scenarios one can expect that the Oct4-initiated invasive phenotype (EMT) or Oct4-silenced EMT may be dictated and tightly regulated by endogenous Oct4 expression.

### Role of Oct4 in drug resistance

As a regulator of pluripotency and self-renewal, it is believed that Oct4 plays a crucial role in the survival of a population of CSCs with drug resistance phenotype [60]. This has been supported by a study in liver cancer cells, where Oct4 over expressing cells were found to be more resistant to cisplatin and doxorubicin treatment compared to control cells both in vitro and in vivo [61]. In oral cancer, Oct4 along with Nanog was shown to be significantly expressed in cisplatin resistant patients [62]. Treatment of oral cancer cells with cisplatin resulted in a population of resistant cells enriched in stem/progenitor cells which displayed increased migratory and invasive capabilities both in vitro and in vivo [62]. This suggests that cancer cells that express Oct4 and survive treatment with cisplatin could develop into a heterogeneous population of differentiated cells that have the increased ability to become metastatic. In support of this proposal, drug resistant prostate cancer cell lines have been shown to have enhanced expression of Oct4 and several target genes (MIDI, MYB, IL1RN, RPS27 and CUGBP2) [60]. These cells exhibited enhanced invasive potential by in vitro assays and tumorigenic potential by in vivo mouse xenograft models [60]. Knocking down Oct4 expression by specific small hairpin (sh) RNA attenuated the growth of drug-resistant cells in vitro and in vivo, suggesting that Oct4 expression in cancer cells not only plays an important role in tumorigenesis but is also essential for acquiring/maintaining a drug-resistant phenotype.

### Evidence of Oct4 in normal ovaries

The literature on human ovarian tissues is limited due to the difficulties in obtaining normal ovaries for research. Scraped human surface epithelium is commonly used to study the biology of epithelial ovarian cells [4,7]. Recent studies have shown the existence of adult human ovarian stem cells in the ovarian surface epithelium of postmenopausal women and women with premature ovarian failure [17,63]. These women had no oocytes or follicles. The ovarian surface epithelium of these women expressed small round bubble-like stem cells with expression of early embryonic developmental markers such as SSEA-4, Oct4, Nanog, Sox-2 and c-kit [17]. In cell culture some of these cells grew in size into oocyte-like cells which expressed Oct4, c-kit, VASA and ZP2 transcription factors specific for early oocytes [17,64]. Later studies have shown the presence of two distinct populations of stem cells in adult mammalian ovarian surface epithelium (including rabbit, sheep, monkey and menopausal women), the very small (1–3 μM) embryonic-like stem cells (VSELs) which are quiescent and pluripotent (PSCs) and express Oct4, Nanog, Sox2, telomerase (TERT) and signal transduction and activation of transcription factor 3 (STAT3) [64]. These small PSCs in culture undergo spontaneous differentiation into larger (4–7 μM) oocyte-like cells, parthenote and embryoid-like structures with neuronal and mesenchymal phenotypes [64-66]. These studies confirm the presence of putative stem cells in the ovaries of adult and older women and have important implications for the treatment of infertile women in the field of reproductive medicine [67].

### Evidence for Oct4 in EOC

The current literature on the expression and role of Oct4 in EOCs is relatively sparse, with the transcription factor primarily being used as marker to detect CSC-like populations in CSC-enriched ovarian cancer cell lines and tumors [10]. The expression of Oct4 was first described in an ovarian dysgerminoma, a tumor of the ovary that is composed of primitive, undifferentiated germ cells [68]. However, these authors failed to detect the expression of Oct4 in other non-germ ovarian tumors such as granulosa cell tumors, Brenner tumors, serous and endometrioid adenocarcinomas and ovarian stromal carcinomas. Recently, Oct4 expression has been described in immature teratoma of the ovary [69], in Fallopian tube epithelium and serous and mucinous epithelial ovarian tumors of different histological grades using immunohistochemical analysis [70]. In this study, Oct4 expression was shown to be significantly increased from normal ovarian surface epithelium/Fallopian tube epithelium to benign/borderline tumors to high grade serous carcinomas, suggesting that the expression of Oct4 is associated with the initiation and progression of serous ovarian cancer [70]. However, this study found no significant difference among normal, benign, borderline and malignant tumors in the mucinous group, and did not study the endometrioid and clear subtype of EOC [70]. The differences in the expression of Oct4 between serous and mucinous EOC may be due to the differences in the genetic makeup of serous and mucinous subtypes of ovarian tumors [71]. Therefore, while the study included a substantial number of human specimens (495 cases, including 35 normal Fallopian tube samples and 40 normal ovaries) no clear distinction of Oct4 expression could be obtained between the different histological sub-types of ovarian tumors. A more recent study on one case report has demonstrated the presence of SSEA-4, Sox2, VASA and ZP2 positive oocyte-like cells on the ovarian surface epithelium of women diagnosed with serous papillary adenocarcinoma [72]. The authors in this case report suggest an association between the pathological condition of serous papillary adenocarcinoma and the presence of primitive oocyte-like cells which may have persisted from foetal period of life of that particular patient or potentially may have developed from putative stem cells (VSELs) of ovarian surface epithelium [67].

The first involvement of Oct4 in EOC stem cells was demonstrated after a single tumorigenic clone was isolated from the ascites of a patient with advanced EOC using serial dilution [73]. This clone demonstrated self-renewal characteristics by forming spheroids in culture and displayed differentiation properties by forming multi-cellular colonies in agar. Furthermore, on serial xenograft implantation, isolated clones continued to establish tumors in nude mice similar to primary human EOC tumors. In subsequent studies, CSCs have been isolated from ovarian tumors and cell lines based on their abilities to differentially efflux the DNA binding dyes, commonly known as side population by flow cytometric pattern [74,75]. This population of stem cells displayed the classical stem cell property in tumorigenicity assays, had an enhanced expression of Oct4, and were resistant to chemotherapy. Side population cells extracted from an ovarian cancer cell line were found to be enriched for the ATP-binding cassette (ABC) transporter (ABCB1) and histone methyltransferase (EZH2 a member of polycomb family with stemness property) and Oct4 after chemotherapy treatment [76]. In the same context, side population enriched cells have been isolated from the ascites of ovarian cancer patients and these have shown an enhanced stemness profile compared to non-side population cells [76]. In addition, gene expression analysis have shown that the side population cell signature was enriched in patients with early recurrence (1–12 months) compared to those with a later (13–24 months) recurrence [77]. Hence, the expression of Oct4 in the side population of ovarian cancer patients may have an important clinical application.

In recent studies several cell surface and non-surface markers have been used to isolate ovarian CSCs. CSCs in these studies have been isolated depending on the distinct pattern of surface markers (i.e. CD44, EpCAM, CD133, CD117, Thy1, CD24) [50,78-80], and non-surface markers (i.e. aldehyde dehydrogenase activity) [81] The CSCs sorted on the basis of these markers have shown the potential to have ‘CSC characteristics’ (ability to self renew, resistance to therapy, develop tumors in very small numbers ~100 cells, etc.), and almost all of them had relatively high expression of Oct4. A recent study has demonstrated the combined expression of Oct4 and Lin28 in ovarian tumors which correlated that with advanced tumor grade [82]. They also demonstrated that the repression of Oct4 together with Lin28 in ovarian cancer cell lines by RNA interference reduced the survival of cancer cells. The most recent study from our group has demonstrated enhanced mRNA expression of invasive and CSC-like markers (EpCAM, CD44, STAT3, Oct4, MMP2 and MMP9) in the ascites-derived tumor and stromal cells isolated from the ascites of chemoresistant versus chemonaive patients [83]. These studies suggest that over expression of Oct4 may be one of the defining features of ovarian cancer stem cells which may regulate cancer progression, drug resistance and recurrence. Hence, Oct4 may be a promising target for therapy in EOC.

### Correlation of Oct4 with Nanog in the context of stem cell biology

Like Oct4, Nanog is also essential for the maintenance of embryonic stem cell fate [84]. Nanog transcript first appears at the ICM of blastocyst after compaction, and is no longer detectable at implantation [85], while Oct4 is expressed prior to compaction in all blastomeres [86]. The expression of both Nanog and Oct4 remains restricted to epiblast as embryonic development progresses [15]. Although both Oct4 and Nanog have independent roles in different cell types, a part of their function in pluripotent cells is driven by a synergistic interaction that drives the transcription of target genes [87,88]. A recent study has demonstrated a cooperative interaction between Nanog, Sox-2 and Oct4 by identifying a composite sox-oct cis-regulatory element within the Nanog proximal promoter [89]. Using chromatin immunoprecipitation, this study showed that Oct4 and Sox-2 bind to the promoter region of Nanog in living mouse and human ESCs, and specific knockdown of Oct4 and Sox2 mRNA by RNA interference reduces Nanog promoter activity to almost that of the background levels, suggesting a genetic link between the pluripotent activity of Nanog promoter and the levels of Oct4 and Sox-2 [89]. In another recent study, endogenous Oct4 and Nanog have been shown to interact and form multiple repression complexes to control gene expression in mouse ESCs, suggesting that these two essential transcription factors associate with unique repressors complexes on their target genes to control the fate of ESC [87].

The pluripotent potential of Nanog along with Oct4 has been evidenced by the expression of these transcription factors in VSELs in adult tissues [16]. Mobilization of VSELs expressing Nanog and Oct4 into peripheral blood has been observed in patients with acute myocardial infarction [90] and acute burn injury [91], suggesting the pluripotent potential of these cells in tissue repair. However, how these two embryonic markers contribute to the development of tumor is still not clearly understood. It has been suggested that epigenetic changes/mutations in the genes that maintains the quiescence of VSELs could potentially lead to tumor formation [18]. Therefore, it will be important in future studies to investigate whether the genomic imprinting pattern differs between VSELs isolated from normal versus tumorigenic populations. In this context, co-expression of Oct4 and Nanog in heptocellular [92], pancreatic [93] and oral [58] cancers has been predictive of a worse clinical outcome. Both Nanog and Oct4 in two independent studies have been shown to be highly expressed in ovarian carcinomas [69,94].

### Confusion with Oct4 Isoforms and problems in analysing data from current literature

**Figure 1:**
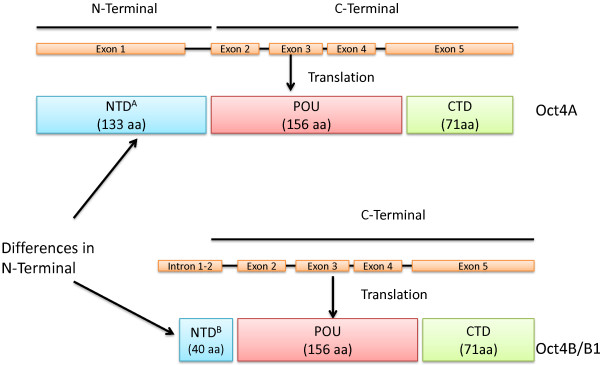
**A schematic diagram representing the human Oct4 isoforms.** Both Oct4A and Oct4B share identical exons 2–5. The differences between the two isoforms lie in exon 1. The self-renewal and pluripotent properties of Oct4 encoded in exon 1 (Adapted from [97] and [96]).

The POU family transcription factors regulate genes containing an octamer motif (ATGCAAAT) in their promoter or enhancer regions [95]. The human Oct4 gene is located on chromosome 6 and consists of five exons. Oct4 encodes for three main variants generated by alternative splicing known as Oct4A, Oct4B and Oct4B1 [96] (Figure 1). At the nucleotide level, both Oct4A and Oct4B are identical in exons 2–5, the differences however lie in exon 1 [97]. Exon 1 is missing in the truncated Oct4B and it specifically consists of exon 2a. OctB1 is identical to Oct4B except it has an additional exon 2c [96]. Human Oct4A and Oct4B are composed of 360 and 265 amino acids respectively and the last 225 C-terminal amino acids are identical in both splice variants [96] (Figure 1). The protein product of OctB1 has not been identified yet. An in-frame stop codon TGA is located in the additional exon 2c of Oct4B1 which is spliced out in Oct4B mRNA [96]. Hence, Oct4B1 cannot encode the full length Oct4B-265 product.

Oct4A is specifically expressed in the nucleus of ESCs, human somatic stem cells, somatic tumor cells and at a basal level in some adult stem cells [96]. The functional protein for Oct4A has not been reliably detected in the non-pluripotent cells, and it is still not clear if the basal expression of Oct4A in non-pluripotent cells endows any biological function. However, a high expression level of Oct4A protein is found in pluripotent cells [96].

Oct4B is expressed at low levels in human somatic stem cells, tumor cells, adult tissues as well as pluripotent cells. The expression of Oct4B is generally localized to cytoplasm [98], and currently there is no evidence to suggest that the Oct4B isoform may be involved in the generation of iPSC. Oct4B has been shown to play a role in the stress response [96], and more detailed biological studies are need to characterize this transcription factor further. However, Oct4B1 has been associated with stemness [96], and further investigations on Oct4B1 are also needed to establish its role in stem cell biology. In spite of this variability and differences in the biological functions of Oct4 isoforms, most studies in the literature do not discriminate between Oct4A, Oct4B or OctB1 at the protein or RNA levels [97]. The fact that the three isoforms are identical at the C-terminal end of the splice variants increases the risk of obtaining false positive signals at the protein and mRNA levels. Immunohistochemistry and immunofluorescence techniques can discriminate between Oct4A and Oct4B by the differences in the nuclear and cytoplasmic localization. However, comparison of the isoforms by Western blot and fluorescence activated cell sorting cannot discriminate between the isoforms by product size or differences in the localization of the fluorescence [97]. Therefore, it is vital to chose isoform- specific antibodies to interpret results relating to stemness. In a similar fashion, much of the data available on Oct4 expression at the RNA level should be interpreted with caution due to possible false positive artefacts which may result from false amplification of the transcripts resulting from improper primer design. To add to the complexity of Oct4 isoforms, six known Oct4 pseudogenes have recently been described [97]. Due to the high homology of pseudogenes to their parental genes, the possibility of amplifying pseudogene-derived PCR product is very high [97]. Hence, to elucidate the expression pattern and the biological functions of Oct4A gene in the context of cancer stemness, it is important to discriminate between the isoforms and pseudogenes of Oct4A [96,97].

### Preliminary data on the expression of Oct4A in EOC chemoresistant cells

#### Human ethics statement

Ascites was collected from patients diagnosed with advanced-stage serous ovarian carcinoma, after obtaining written informed consent under protocols approved by the Human Research and Ethics Committee (HREC # 09/09) of The Royal Women’s Hospital, Melbourne, Australia.

**Figure 2:**
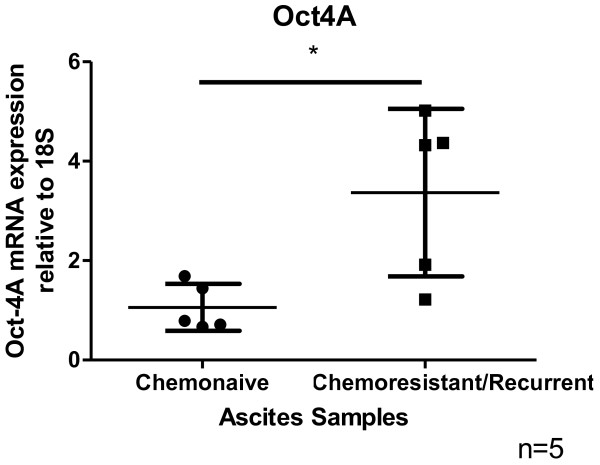
**mRNA expression of OCT4A in isolated cells obtained from chemonaive and chemoresistant ovarian cancer patients.** Ascites cells were isolated as described previously [10]. RNA extractions, cDNA synthesis and quantitative determination of mRNA levels of Oct4A were performed as previously described [99]. Sense and antisense primers were designed against published human sequences for Oct4A (Entrez Gene ID 5460, approved symbol POU5F1): forward- CTCCTGGAGGGCCAGGAATC; reverse- CCACATCGGCCTGTGTATAT; 18S (Entrez Gene ID 100008588, approved symbol RN18S1) forward-GTAACCCGTTGAACCCCATT; reverse-CCATCCAATCGGTAGTAGCG. Gel extraction of PCR products was performed using the QiaEX II Agarose gel extraction Kit (Qiagen Australia), as per the manufacturers’ protocol and quantified using the ND-1000 Nanodrop spectrophotometer (NanoDrop Technologies Inc Wilmington, DE, USA). Sequences and products were verified as described previously [99]. Results are expressed as the difference between the log2 transformed ΔCt values of the gene of interest to that of housekeeping gene (18S) ±SEM of five independent samples performed in triplicate. *P<0.05, significantly different in recurrent versus chemonaive ascites samples.

We have previously demonstrated that cisplatin treatment of ovarian cancer cells (primary, ascites tumor cells and cell lines) treated with cisplatin results in a population of residual cells with enhanced stemness including increased expression of Oct4 compared to untreated cells [10]. We now provide evidence to suggest that the mRNA expression of Oct4A was enhanced in the cells isolated from the ascites of recurrent EOC patients compared to cells isolated from the ascites of chemonaive patients (patients with primary carcinoma who have not undergone any treatment). The recurrent patients previously received combinations of chemotherapy consisting of paclitaxel, carboplatin and drugs such as doxorubicin, gemcitabine, docetaxel, cyclophosphamide and topotecan after each recurrent episode. These patients were diagnosed with recurrent disease 6–20 months after first line of chemotherapy. Oct4A expression was significantly enhanced in the ascites cells of recurrent patients compared to chemonaive patients (Figure 2).

**Figure 3:**
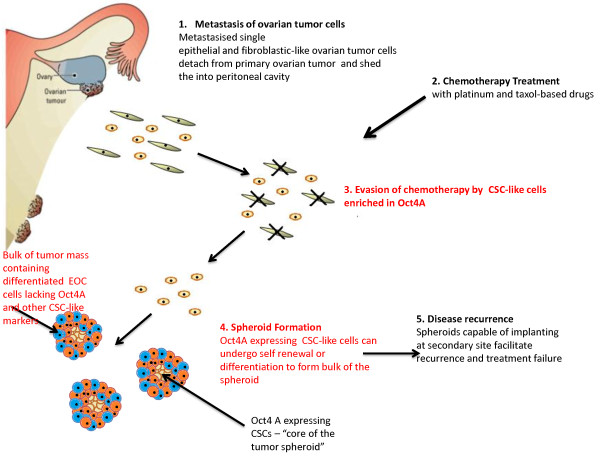
**A model of Oct4A-mediated ovarian cancer evolution and progression in the ascites microenvironment.** During the course of ovarian cancer progression a shedding of tumor cells into the peritoneum occurs. Here tumor cells survive as cellular aggregates/spheroids where CSC-enriched core cells of the spheroids serve as a niche for regenerating cells. During chemotherapy treatment the bulk of the differentiated tumor cells on the periphery of the spheroids are eradicated leaving behind CSC-enriched core tumor cells. These cells facilitate the self-renewal of chemotherapy surviving residual cells resulting in tumor recurrence.

Interestingly, the enhanced expression of Oct4A in the ascites cells of recurrent patients can be related to the biological actions of Oct4 occurring in developing embryos [13]. A developing embryo relies on a fundamental switch from an undifferentiated to differentiated state of the inner cellular mass (ICM) of a blastocyst [28]. While Oct4 expression is uniformly expressed across all cells of the ICM, a loss of Oct4 expression in these cells results in spontaneous differentiation to form cells of the outer protective trophectoderm structure [13,28]. This loss is known to be imperative for the formation of definitive structures of a developing embryo such as the outer protective trophectoderm surrounding the ICM and reinforces the role of Oct4 as a pluripotent regulator. Interestingly, this scenario of trophectoderm formation of the developing embryo can be applied to events that appear during the course of EOC recurrence involving CSCs. In ovarian cancer spheroids, the ICM would represent the core of a metastasised tumor spheroid containing chemoresistant CSC-like cells that evade chemotherapy. Following chemotherapy treatment, these Oct4A expressing residual CSC-like cells would be capable of undergoing self-renewal and differentiation leading to reformation of ascites tumor masses (Figure 3). If confirmed, this model would potentially provide the link between CSCs and chemoresistance in ovarian cancer.

### Oct4A as a therapeutic target for EOC

Oct4A is expressed at relatively low levels in normal somatic tissues compared to their respective tumorgenic cells, suggesting that targeting Oct4A may be a good strategy to disable CSCs in EOC. Eliminating the self-renewing and pluripotent ability of CSCs could prevent EOC tumor progression and eradicate chemoresistance and subsequent recurrence. While obvious targeting methods include inhibiting the upstream targets of Oct4 such as WNT, AKT and TGFβ [100], a relatively newer proposed method would be to target specific miroRNAs (miRNA) responsible for regulating Oct4A expression in ovarian cancer progression and chemoresistance.

### MicroRNAs associated with Oct4

MicroRNAs (miRNAs) approximately 21–23 nucleotides long are a group of non-coding RNAs that can regulate gene expression by degrading their target messenger RNAs (mRNAs) by binding to the complementary sequences found in the 3’-untranslated region (UTRs) of target mRNAs [101]. This result in the modulation of a cascade of cellular functions, including those related to ESC self-renewal/differentiation [102]. Recently, deregulation of some miRNAs has been implicated in a number of human cancers where they can act as either tumor suppressors or as tumor oncogenes [102]. Interestingly, an increasing amount of evidence also suggests that miRNAs play a role in self-renewal and differentiation with only a few studies describing a role of miRNAs in reprogramming of somatic cells [103], and in the regulation of cancer stem cells [101,104].

The *let7* family is one of the most extensively studied and well understood of all miRNAs involved in carcinogenesis, and has emerged as an important regulatory factor in a range of cancers including ovarian cancer [105]. Upregulation of *let-7* is a prominent feature of ESC differentiation, and ESCs are characterised by a striking down-regulated *let-7* expression, which is dominantly expressed in most differentiated cells in the vast majority of tissues [105]. Lin28 on the other hand is highly expressed in ESC and cancer cells and has been demonstrated to be down regulated during differentiation [82]. A high Lin28/low *let-7* signature is common in ESC, iPSC and CSC [106]. A recent study has shown that overexpression of *miR-125b* in hESC resulted in the upregulation of the early cardiac transcription factors, GATA4 and Nkx2-5, and accelerated the progression of hESC-derived myocardial precursors to an embryonic cardiomyocyte phenotype [107]. By using an *in silico* approach, *let-7*, Lin28 and Oct4 were identified as targets of miR-125b, suggesting that the manipulation of *miR-125b* -mediated pathways may be useful for reprogramming ESC to different lineages. In this context, *let-7*, *miR-125*. *miR-9* and *miR-30* have been shown to repress Lin28 expression in ESC and cancer cells [108].

Since 2006, a few studies have shown that the miRNA profile is different in normal ovaries compared to primary and recurrent ovarian tumors [109]. *Let-7a* and *miR-200* families have been shown to be deregulated in ovarian pathogenesis [109]. Decreased expression of *let-7* has been associated with the mesenchymal aggressive phenotype (C5) of high-grade serous ovarian carcinoma [110]. Down regulation of *let-7* has also been associated with cisplatin and taxol resistance [111,112], which suggests that restoring the expression of *let-7* may be a useful therapeutic option overcoming drug resistance. The combined expression of Lin28 and Oct4 has been demonstrated in high-grade ovarian carcinomas [82]. Viral delivery of *let-7* has also been shown to suppress the tumor growth in a mouse model of lung adenocarcinoma [113]. These studies suggest that increasing the expression of *let-7* may be another novel therapeutic option to minimise/eradicate chemoresistant recurrent ovarian tumors.

### Oct4 in transdifferentiation

By introducing specific transcription factors, it is possible to induce cells into an alternative fate through transdifferentiation. Transdifferentiation of mouse embryonic fibroblasts into functional cardiomyocytes by overexpressing Oct4, Sox2, Klf4 and c-Myc under defined cardiac cell culture conditions has recently gained attention [114]. Hence, by modifying culture conditions somatic cells can be induced to undergo transdiffferentiation into cells of other lineages by introducing an iPSC cocktail which includes Oct4 [115]. In this context, the use of specific unsaturated fatty acids, such palmitic, oleic and, linoleic acid that can trigger adipocyte differentiation in human cancer cell lines, including ovarian cancer is worth considering [116] This study demonstrated massive production of lipid droplets and up regulation of the adipogenic nuclear regulator PPARγ, which belongs to the Peroxisome Proliferator-Activated Receptor (PPARs) superfamily. As PPAR γ is over expressed in ovarian carcinomas [117], this adipogenic transdifferentiation may be a feasible option in combination with chemotherapy or post-chemotherapy in a certain sub-set of ovarian carcinomas. In addition, PPAR γ ligands, drug such as pioglitazone, troglitazone and ciglitazone have been shown to modulate PPAR γ activity by effecting the proliferation of ovarian cancer cells [118]. These differentiation strategies represent promising non-cytotoxic method of decreasing tumor burden, but how such an approach will impact on the Oct4A-enriched CSC pool and activity yet remains to be determined. We suggest that these transdifferentiation studies can be extended to ovarian cancer, and that Oct4 is likely to be a key player.

## Conclusion

The role of Oct4 in EOC tumorigenesis is still not well defined. However, while Oct4 appears to be essential during embryogenesis and reprogramming of somatic cells, enhanced or overexpression of Oct4A may be a prime factor for EOC initiation, progression and recurrence. Its expression is enhanced in high-grade serous ovarian carcinomas and consistently associated with CSC-like populations which are believed to be responsible for recurrent and resistant disease. Therefore, unless methods to directly target these specific Oct4 expressing populations can be found, it is believed that this resistant and recurrent cycle of tumor growth after debulking surgery and initial chemotherapy will continue, contributing to the tumor burden which leads to patient deaths.

## Competing interests

The authors declare that they have no competing interests.

## Authors’ contribution

CS and NA conceived the idea, designed and wrote the manuscript. MQ and JFK edited the manuscript. All authors read and approved the manuscript.
